# Can you un-ring the bell? A qualitative study of how affect influences cancer screening decisions

**DOI:** 10.1186/s12885-017-3596-7

**Published:** 2017-09-13

**Authors:** S. Michelle Driedger, Gary Annable, Melissa Brouwers, Donna Turner, Ryan Maier

**Affiliations:** 1 Community Health Sciences, College of Medicine, Faculty of Health Sciences, University of Manitoba, S113-750 Bannatyne Avenue, Winnipeg, MB R3E 0W3 Canada; 2 Department of Oncology, McMaster University, Juravinski Site, 60 (G) Wing, 711 Concession Street, Hamilton, ON L8V 1C3 Canada; 3 Population Oncology, CancerCare Manitoba, 675 McDermot Avenue, Winnipeg, MB R3E 0V9 Canada

**Keywords:** Chronic disease, Prevention, Guidelines, Prostate cancer, Breast cancer, Mammography, Prostate specific antigen, Decision making, Uncertainty, Affect

## Abstract

**Background:**

The belief that early detection is the best protection against cancer underlies cancer screening. Emerging research now suggests harms associated with early detection may sometimes outweigh the benefits. Governments, cancer agencies, and organizations that publish screening guidelines have found it is difficult to “un-ring the bell” on the message that “early detection is your best protection” because of its widespread communication and enduring resonance. This study explores affective factors—and their interplay with relevant analytical factors—in public/laypersons’ decision making about cancer screening.

**Methods:**

A total of 93 people (47 men, 46 women) attended focus groups about, respectively, prostate cancer screening and breast cancer screening in two Canadian cities.

**Results:**

Affective factors were a major influence on many focus group participants’ decision making about cancer screening, including fear of cancer and a generalized enthusiasm for prevention/screening, and they were often inspired by anecdotes about the cancer experiences of family and friends. Affect also existed alongside more analytical factors including assessments of reduced risk in the management of any cancer diagnosis if caught early, and, for men, the belief that an unreliable test is “better than nothing,” and that men deserve prostate cancer screening because women have breast and cervical cancer screening. Affective factors were particularly noticeable in the sub-groups most supportive of screening and the “early detection” message: older women who felt that mammogram screening should begin at age 40 rather than 50, and older men who felt that prostate cancer screening should be expanded beyond its current unorganized, opportunistic usage. In contrast, younger participants displayed less affective attachments to “early detection” messages and had greater concerns about harms of screening and were more receptive to nuanced messages informed by evidence.

**Conclusion:**

Policymakers attempting to communicate more nuanced versions of the “early detection” message need to understand the role of affect alongside other judgments brought into laypersons’ decision making processes and anticipate how affective responses to their messages will be shaped, transformed, and potentially subverted by external forces beyond their control. Particularly overt external factors are campaigns by cancer advocacy organizations actively promoting breast and prostate cancer awareness and screening to younger women and men using affectively-charged messages.

**Electronic supplementary material:**

The online version of this article (10.1186/s12885-017-3596-7) contains supplementary material, which is available to authorized users.

## Background

The widespread promotion of the message that early detection and treatment is the best protection against cancer has great intuitive resonance. Recent research, however, suggests the harms associated with early detection for some cancers may sometimes outweigh the benefits [1–3]. Governments, cancer agencies, and organizations that publish screening recommendations that have made policy changes incorporating this new evidence have faced considerable challenges from the public, cancer advocacy organizations, and specialist groups, and are finding that it is difficult to “un-ring the bell” on the message that “early detection is your best protection.” Drawing from qualitative focus group data, this paper explores affective factors – and their interplay with relevant analytical factors – in patient decision making about cancer screening.

### Affect in health care decision making

Dual-process theories of risk perception and/or decision making [4–6] propose that humans process risk information through two parallel pathways, described variously as *intuitive* and *analytical* [7–9]. Intuitive information processing has a strong *affective* basis [10]. Affective responses – which include emotions (relatively intense affective states) and moods (milder, more diffuse states) – are generally automatic and pre-conscious. The importance of affect and its role in a dual-pathway schema in decision-making has been supported by an increasing body of research over the last several decades, including prominent work by Zajonc, McAllister, Finucane, and Slovic, among others [10–17]. In the affective domain, people may encounter a stimulus and immediately link it to relevant and established feelings or emotions (i.e. positive or negative) they associate with it, which thereby motivates and shapes their response [18, 19]. Slovic and colleagues call this process the “affect heuristic” – a mental shortcut for making judgments about risks which is especially useful when the risk in question is complex and uncertain [10]. They also demonstrate that as people link an activity to more positive feelings they have about it, they will tend to judge associated risks as lower – and vice versa [10].

By contrast, the analytical pathway is characterized as deliberative, calculative, slower, and requiring cognitive effort. When one is confronted with a stimulus, this pathway is reserved for the processes of reasoning, logic, weighing of risk probabilities and recalling details about past experience, and conjecture [16]. The dual-process framework holds that one pathway may predominate or pre-empt the other when someone encounters a situation [20, 21], yet often the two are very intertwined, with one providing support or guidance for the other [11, 16, 22]. While dual-pathway models share the same relative form, they differ slightly in terms of their thematic content – though not substantially. For example, the model conceptualized by Siegrist, Earle, and colleagues also contains a dynamic and interactive intuitive/analytical dichotomy [23–25]. Their Trust, Confidence, and Cooperation (TCC) model proposes that when one is considering whether or not to accept a risk or to cooperate with given recommendations, one can make intuitive judgments of trust towards another person/institution (based on assessments of similar values, which is often grasped on more emotional levels), and one can evaluate the risk involved based on past experience, or the knowledge one has of the risk. In the context of cancer screening, it is likely that intuitive feelings and emotions and evaluations of trust, as well as analytical or rational assessments of risk and personal experience/knowledge all play varying roles in influencing decision-making and perceptions of risk.

Cancer, being perhaps the most feared of modern diseases, is certainly saturated with affect, and is often characterized by battle/war metaphors (enemy, fight, destroy, triumph). The mere mention of cancer generally inspires an immediate sense of fear, anxiety, uncertainty, and dread (so much so that the word used to be, and often still is, avoided, or recast as “the Big C”) [26]. These feelings may have built up to varying degrees (or not) over the course of someone’s life especially if cancer has affected some part of their life (i.e. whether themselves or family and friends). Discussion of cancer or testing may then cue someone to reflexively react with associated affective attachments that they have. However, previous research about laypersons’ decision making about cancer screening has devoted greater attention to analytical processing and less to intuitive processing [27, 28]. On the other hand, studies have begun to show that intuitive factors like affect (e.g. fear) have a substantial, and, at times dominant, role in influencing screening behavior [29]. In fact, affective responses may be so strong that they may over-ride the analytical pathway if there is a conflict between the two. A study by Farrell, Murphy, and Schneider has shown that fear of prostate cancer caused men to ignore evidence unfavorable to screening and to maintain that the benefits of screening outweighed the risks [30]. While the intuitive or affective domain will be the primary focus of this paper, we will also show the ways in which that pathway interacts with other analytical or cognitive processes (e.g., judgments of risk or more analytical knowledge processes) when people make decisions about cancer screening.

Health risk communication messages often aim to induce affective responses. They may induce affect *integrally* (directly through the message) and *incidentally* (unintentionally, e.g., how the message is presented in media coverage). Because incidental affect is primarily shaped by external factors beyond the control of health risk communicators, communicators may have difficulty controlling messages that induce affect incidentally [31].

There has been a growth in Canadian news media portrayals of cancer. In these stories, fear is one of the most frequently identified themes that is often linked to the necessity and benefits of early detection [26]. Cancer screening programs are grounded in the belief that early detection is the best protection against dying of cancer, using clinical guidelines and evidence to inform at which age or circumstances different screening programs should be implemented on a population basis. This “early detection” message is present in many of the public information materials published by cancer agencies and cancer advocacy organizations [32, 33]. Cancer advocacy organizations frequently embed this messaging with affective cues and stimuli alongside selected evidence so that audiences will be more receptive of their views and recommendations [34–38]. For instance, they may attempt to influence the screening behavior of young people by showing pictures and sharing anecdotes of young people who have had cancer and survived or have died. The “early detection” mantra is almost ubiquitous in health messaging about cancer, and carries with it affective tags that relate to the fear about cancer, as well as to the hope for a desirable outcome if a cancer is discovered early enough to treat. It portrays an ethic of responsibility wherein it is in society’s best interest to catch and treat all cancers as soon as possible, and that doing so demonstrates an alignment of values between the public and official health system stakeholders.

### Benefits and harms of cancer screening

Indeed, “early detection” messaging has been communicated so effectively that studies in the U.S., Europe and New Zealand have found that most people radically overestimate the mortality reduction benefit that results from mammography and PSA screening [39–41], in spite of new evidence challenging this message [3, 42]. There is increasing evidence that some screening tests are unreliable, producing high rates of false-positive results that may lead to unnecessary and potentially invasive and risky follow-up testing (e.g., biopsies) along with the psychological anxiety associated with the possibility that one has cancer. Other research suggests that treatment of cancers detected through screening may not always be necessary or beneficial (i.e., overdiagnosis and overtreatment) [43]. The estimation of overdiagnosis and overtreatment is controversial [44, 45], and tests to distinguish harmless or more slow-growing cancers from those that are deadly remain elusive [46].

Previous research on affect and cancer screening has tended to focus on specific affective responses (e.g., fear, worry) [47–50]. There is, however, a growing body of broader examinations of the role of affect and analytical judgments in individuals’ decisions about cancer screening [29, 30, 51, 52]. For example, one study found that most men who were presented with information about the risks of prostate cancer screening interpreted the information as being unfavorable to screening, yet still felt the benefits outweighed the risks and planned to continue to be screened. Fear of cancer was a factor, but other affective influences included anecdotes about the cancer experiences of friends, family or celebrities, a general distrust of statistics, and overall enthusiasm for prevention [30]. Another study found that laypersons’ affective associations with colonoscopy screening fully mediated the relationship between study participants’ analytical judgments about colonoscopy and their intentions to be screened [29]. Research has also found that members of the public have high tolerances for screening harms [53, 54]. One study of women’s attitudes towards false-positive mammogram results found that most participants were prepared to tolerate 1000 or more false-positives to save one woman’s life. Women who had experienced false-positives expressed tolerances as high as women who had not [54].

### Breast and prostate cancer screening in Canada

The most comprehensive set of Canadian cancer screening guidelines are published by the Canadian Task Force on Preventive Health Care (CTFPHC). The task force’s current breast cancer screening guidelines were published in 2011 and recommend that average-risk women aged 40 to 49 years should not have routine mammograms because the benefits are small and outweighed by harms [1]. That recommendation was particularly controversial. The task force’s previous guidelines had never recommended that 40 to 49 year old women *should* be screened; in 1994 it recommended against screening this age group [55] and in 2001 concluded that the existing evidence was insufficient to recommend for *or* against screening [56]. Nevertheless, the shift from 2001’s uncertain conclusion to 2011’s definitive recommendation against routine screening for women aged 40 to 49 triggered considerable criticism from breast cancer advocacy organizations and radiologists that this recommendation would needlessly put women’s lives at risk. Against a backdrop of increasingly scarce healthcare resources, such confusing and competing expert opinion can leave the public with the impression that health systems view saving money as more important than saving lives [57].

Organized mammography screening programs exist in 12 of Canada’s 13 provinces/territories [58]. All of them offer screening to average-risk women between the ages of 50 and 69, but there is considerable variation in screening eligibility for women under 50 [59]. Two provinces include women aged 40 to 49 years in their screening programs, while women in this age group in seven provinces and territories can access screening mammograms by self-referral or with physician referral [60]. Three provinces exclude average-risk 40 to 49 year old women from mammogram screening, but physicians can order *diagnostic* mammograms for women under 50 throughout Canada, which likely includes some number of de facto screening mammograms performed outside organized screening programs. Notably, little has changed since the CTFPHC published its revised recommendation in 2011; despite subtle shifts in two provinces/territories that now require physician referral, all provinces/territories that provided screening to women aged 40 to 49 when the new recommendation was published [60] continue to do so [59].

The CTFPHC published revised recommendations on screening for prostate cancer with the prostate-specific antigen (PSA) test in 2014 [2]. The task force recommended that the PSA test should not be used for screening, arguing that the small and uncertain reduction in mortality is outweighed by harms (false-positives, unnecessary biopsies, overtreatment). As with mammography for women aged 40 to 49, this recommendation was met with criticism from prostate cancer advocacy and urologist organizations [61–65].

There are no population-based prostate cancer screening programs in Canada. The patient advocacy organization Prostate Cancer Canada and the Canadian Urological Association promote the use of the PSA test to screen men, but do not recommend its use as a *population-based* screening tool (i.e., the formalized process of inviting everyone within an age group to take part in an organized screening program) [66, 67]. Nevertheless, the test has been widely used in clinical practice for opportunistic screening (i.e., when someone asks for a test or when is it offered by a physician) of Canadian men since the early 1990s [68, 69]. The cost of a PSA test for screening purposes is covered by the publicly-funded health care plans in seven of Canada’s ten provinces. Three provinces do not cover PSA tests for screening purposes, so asymptomatic men in those provinces who want a PSA test must pay for it themselves [70].

Despite the Canadian task force’s guidelines recommending against PSA screening in men and mammogram screening in women aged 40 to 49, these differences in policy and practice in different Canadian provinces/territories indicate that considerable uncertainty remains regarding the relative benefits, harms, and overall value of prostate cancer screening and breast cancer screening in women under the age of 50. It is within this context that this study explores the interplay of affective and calculative/analytical factors in patient decision making about breast and prostate cancer screening, and the implications for cancer screening policymakers and communicators.

## Methods

An additional file provides a more detailed methods description, with a particular emphasis on the analysis process (see Additional File 1).

### Study design

This research was conducted as part of a study examining uncertainty in decision making about cancer control policy in Canada funded by a grant from the Canadian Cancer Society Research Institute. The University of Manitoba’s Health Research Ethics Board approved the study protocols (H2010:194).

### Participants and recruitment

Women aged 35 to 59 years (*n* = 46) and men aged 45 to 74 (*n* = 47) were recruited by survey research companies for focus groups about, respectively, breast and prostate cancer screening. Participants did not have a history of cancer.

### Data collection

Established protocols for conducting focus groups were followed [71–73]. Ten focus groups about breast cancer (*n* = 5) and prostate cancer (*n* = 5) screening were held in Toronto, Ontario (*n* = 6) and Winnipeg, Manitoba (*n* = 4) in May and June of 2012. The discussions were guided by an instrument (see Additional files 2 and 3) that began with open-ended questions about cancer, tests for detecting cancer, population-based screening, and how participants made decisions about their health. At the mid-point of each meeting, the interviewer gave plain language descriptions of current guidelines for breast/prostate cancer screening, a summary of the relevant research evidence, and a description of remaining uncertainties. These presentations were accompanied by hand-outs given to each participant. Following these presentations, women were asked at what age they thought mammogram screening should begin, and men were asked if they thought there should be an organized prostate cancer screening program. Men in Toronto were also asked if they thought the Ontario provincial healthcare system should cover the cost of PSA tests for screening. Each focus group participant received a $60 honorarium.

### Data analysis

All focus groups and interviews were digitally audio-recorded, transcribed verbatim, and audio-verified. All data were analyzed using NVivo10™. For each data set, SMD, GA, RM and one other staff person developed a codebook of draft codes and operational definitions for surface level content after independently reviewing a sample of transcript excerpts. After comparing their draft coding schemes, they resolved disagreements through consensus, and eventually agreed on the final coding framework that was used to systematically code each line of transcript text into surface descriptive content categories. Multiple coders were required for this and a related study. A sample of transcripts were test-coded to establish inter-coder reliability achieving a 0.91 Kappa score [74], and once achieved, assigned coders subsequently coded all the data. Salient categories emerging from the data included: decision-making factors, emotions, risk/risk groups, finance/monetary issues, age, family/friends, side effects/harms, uncertainty, PSA, mammograms, and government policy. Despite the topic of this paper, surface level descriptive coding did not aim to force-fit transcript text into pre-existing categories of analytical or intuitive thematic nodes, even though there were lines of text that might be descriptively coded as worry (i.e. intuitive) or personal assessment of risk (i.e. potentially analytical). Rather, SMD and GA performed analytical queries to identify key themes in the coded data. The output of each of these queries would result in different groupings of coded data where team members could then undertake a deeper reading of the associated text and develop associated memos per established protocols when using a software like NVivo. This process follows a constant comparative and concept-development approach [75] that eventually structured the analysis of data reported here.

### Presentation of results

Results include quotations to illustrate typical responses and, where relevant, the diversity of perspectives. Each focus group quotation is labeled with a first-name pseudonym, age (in 5-year groupings), and city.

## Results

### Sample characteristics

Table 1 presents the socio-demographic characteristics of the 47 men and 46 women who attended the focus groups. Ages, family incomes, and marital statuses were suitably diverse, but educational attainment skewed to higher levels. In particular, 83% of the women had at least some post-secondary education. Most (28) of the 46 women reported having had mammograms (Table 2). All of those aged 50 years and older had mammograms, while most women under 45 years had not. Approximately half of the women aged 45 to 49 (six of 11) reported having mammograms. Most (30) of the 47 men reported having had PSA tests (Table 3). Men over 50 were most likely to have been tested/screened, but three of the seven men aged 45 to 49 had as well. Seven men, mostly in the 45 to 49 age group, had not had PSA tests. Ten (mostly aged 50–59) did not disclose or were unsure if they ever had a PSA test.

**Table 1:** Focus group participant characteristics

	Men		Women	
	n	%	n	%
Age (years)				
35–39	–	–	9	19.6%
40–44	–	–	9	19.6%
45–49	7	14.9%	11	23.9%
50–54	7	14.9%	7	15.2%
55–59	10	21.3%	10	21.7%
60–64	9	19.1%	–	–
65–69	8	17.0%	–	–
70–74	6	12.8%	–	–
Total	47		46	
Highest Level of Education				
Some high school	5	10.6%	1	2.2%
High school graduate	10	21.3%	7	15.2%
Some post-secondary	7	14.9%	14	28.3%
Post-secondary graduate	25	53.2%	25	54.3%
Total	47		46	
Annual Family Income				
Under $25,000	8	17.0%	6	13.0%
$25,000 to $49,999	13	27.7%	11	23.9%
$50,000 to $74,999	8	17.0%	10	21.7%
$75,000 to $99,999	10	21.3%	12	26.1%
$100,000 and over	8	17.0%	7	15.2%
Total	47		46	
Marital Status				
Single, never married	14	29.8%	13	28.3%
Married or common-law	24	51.1%	22	47.8%
Divorced, separated or widowed	9	19.1%	11	23.9%
Total	47		46

**Table 2:** Have you had a mammogram?

	Yes	No	Totals
35–39 years	2	7	9
40–44 years	3	6	9
45–49 years	6	5	11
50–54 years	7	0	7
55–59 years	10	0	10
Totals	28	18	46

**Table 3:** Have you had a PSA test?

	Yes	No	Unclear	Totals
45–49 years	3	4	0	7
50–54 years	3	1	3	7
55–59 years	5	1	4	10
60–64 years	8	0	1	9
65–69 years	6	1	1	8
70–74 years	5	0	1	6
Totals	30	7	10	47

### Affect in cancer screening decision making

Although none of the focus group questions asked specifically about fear, approximately one-third of the participants (29 of 93) expressed fear about some aspect of cancer or cancer screening. The largest number were afraid of the particular cancer discussed in the focus group (*n* = 14), while others expressed fear of cancer in general (*n* = 3). Other participants expressed fears associated with screening harms (*n* = 13), including discomfort or radiation exposure from mammograms, discomfort from digital rectal examination, fear of false-positive test results, unnecessary biopsies, and/or adverse effects of potentially unnecessary treatment. These two sub-groups (participants who expressed fear of getting cancer and those afraid of screening harms) were almost mutually exclusive—only one participant expressed fear of cancer *and* fear of screening harms. However, while feelings of fear and anxiety were the most common affective factors driving enthusiasm for cancer screening or testing, participants’ strong emotional reactions during the discussions were often intertwined with analytical or rational factors.

#### Breast cancer screening – Women’s focus groups

Amongst the 29 women who expressed clear opinions about the age mammogram screening should begin, most (17) felt it should begin at age 50, which is the current practice in both provinces where the focus groups were held. Most of these women, however, rationally qualified their answers by adding that women under 50 should have the option of accessing screening mammograms if they have a family history of breast cancer or their doctors order mammograms. (In Canada, for women with a family history or if a physician was concerned over symptoms a woman was presenting, a diagnostic mammogram can be ordered outside of the screening program). The other 12 of 29 women who expressed opinions felt that mammogram screening should start at age 40. These women commonly framed their support for early detection with affective responses. These included a positive and immediate feeling of enthusiasm for the benefits of early detection, which were often then linked to practical rationale, such as the potential for improved survivability, keeping families intact, and anecdotes about younger women who had breast cancer.

I think it should be 40. The earlier you do it the better to detect it. (Madeline, 40–44, Toronto)

Women between 40 and 49 are important too. They have younger children. If we can save someone’s life by starting at 40, we should be starting at 40. (Eva, 50–54, Toronto)

I had a girlfriend who passed away who was only 34 when she had breast cancer. I think they should lower the screening age. (Florence, 55–59, Toronto)

Even though some women were more supportive of screening benefits in terms of early detection, others did not unproblematically accept a doctor’s recommendation to be screened if they were not at the appropriate age to be invited into the population based screening program, although she might find it impossible to resist the physician’s recommendation. In the case of one participant, when she turned 40, her doctor recommended she be screened. She resisted going, employing both intuitive (pain, discomfort, fear it may expose unnecessary harms) and analytical assessments (no prior family history, uncertainty about its benefits) in her reasoning:

When I turned 40, my doctor sent me for a mammogram. She told me I would have to go every year. We have no history of breast cancer, any kind of cancer in our family. So I kept putting it off. And she’ll check my record and tell me “you did not go for the mammogram.” [I did finally go] and I had a very, very painful experience with it. […] I heard some people say it’s good to detect cancer. Some people said you will get cancer by doing that, the procedure, the way it’s been done. It’s squeezing your breasts, and I don’t know. I have mixed feelings about it. That’s why I still have to do one from last year. And I haven’t gone to do it yet. I keep putting off the appointment. I just don’t want to do it (Noelle, 45–49. Toronto)

Although in the end, this participant, who at the time of the focus group was 47, had undergone at least four mammograms, she remained uncertain about the benefits of following her physician’s clinical recommendation. What is noteworthy, is later in the focus group, this same participant struggled with her stated preference for the age at which screening should start. When the question was asked of all participants, she was in the group of people that initially indicated that screening should start at 50. However, under the pressure of stronger voices in the room, she switched her answer to 40, but with the added caveat: “it should be my choice to have one”. Despite her preference for ‘choosing’ to go (or not), she feels considerable pressure from her clinician to be compliant, because when the physician finds no mammography result they simply present her with “another requisition” to go.

Overall, however, age differences were apparent in who was more enthusiastic about screening as well as the perceptions of screening harms. Interestingly, older women were more likely than younger women to argue for beginning to screen women at 40 (Table 4).

We are not all the same, everybody is different. You can’t say, ‘Oh you’re fine until you’re 50’ and [at] 50 that happens and you’re too late. (Mel, 50–54, Toronto)

Conversely, most women who felt that screening should not begin until age 50 were women under 50. In two focus groups restricted to younger women (35–49 years), only one participant expressed a clear opinion for screening women younger than 50.

With all the things that I hear [about risks], I don’t want to do this screening. I don’t think I will go until my physician sends me that letter in the mail when I hit 50. (Beth, 35–39, Winnipeg)

**Table 4:** Age (in years) to start mammogram screening

	40	50	Unclear	Totals
35–39 years	1	5	3	9
40–44 years	2	4	3	9
45–49 years	3	3	5	11
50–54 years	3	2	2	7
55–59 years	3	3	4	10
Totals	12	17	17	46

Amongst the eight women who expressed a clearly positive or negative opinion about their tolerances for false-positive mammogram results, six were more analytical in their reasoning, and calculated that the harms exceeded the benefits, while two were comfortable with the potential harms of screening as long as it would result in saving one woman’s life and spare their family the emotional cost. While stating her opinion, one of the latter participants spoke in affectively laden terms (while showing empathy) and pointed to a hand-out showing the Canadian Task Force on Preventive Health Care’s calculations (see Additional file 2) of the number of women in the 40 to 49 age group (2108) that would need to have mammograms for eleven years to prevent one woman from dying of breast cancer, as well as numbers of women who would experience false-positive results (690) and unnecessary biopsies (75) [1].

I’m thinking that to prevent one death, if I was one of these unnecessary biopsies, I could have been this one death too. I would undergo these other things and have the false-positives, go through all that in order for one life to be saved, for one mother to still be there. I think it’s worth it. If that was me, or my daughter or my mother, then you think of it in a different way. To me, I would rather have a little bit of a scare and do all this and have one person still be there for their family. (Margaret, 55–59, Winnipeg)

Six women (five under the age of 50) felt the potential harms of screening were too high a price to prevent one 40 to 49 year old woman from dying of breast cancer. One gestured at the graphic depicting the Canadian task force’s calculations and, like Margaret above, invoked affectively-charged language about young women’s personal and family lives, but then employed more rational cost-benefit calculations of risk that emphasized the harms that could come to those women with false-positives rather than the benefit of preventing one breast cancer death.

These are not just little pink women on a page. They’re real women with real lives and real families and friends, and when you create all this extra stress in their lives by having these false-positives, the danger is that stress has its own health effects too. So as much as I want to save this one person, it’s just creating a lot of stress in these other women’s lives. (Louisa, 35–39, Winnipeg)

#### Prostate cancer screening – Men’s focus groups

In the prostate cancer screening focus groups, most (21) of the 32 men who expressed clear opinions about the current opportunistic usage of PSA screening supported the practice. These included men who offered relatively uncritical positive opinions, as well as men who were aware of the PSA test’s unreliability and potential for overdiagnosis and overtreatment, but still felt that screening was worthwhile. Fear of cancer and dying was the main affective factor for men with more positive than critical opinions of PSA screening. At the same time, men also offered more analytical arguments in that, despite the PSA test’s unreliability, in the absence of a better test to screen for prostate cancer, it is “better than nothing”. Similarly, they relied on their previous experience with the PSA test to assess its utility, where they felt that without it, they could be vulnerable to a perceived unnecessary risk of having undiagnosed cancer. They also shared anecdotes about friends and family who had been diagnosed with prostate cancer.

I get tested every year with the PSA, so if that’s the test then I’m happy with the test until there’s something better. (Stephen, 55–59, Winnipeg)

To throw the test away because it’s giving a percentage of false-positives is [like] throwing the baby out with the bath water. (Ernest, 65–69, Winnipeg)

I think the PSA is good because it gives you something to work with. It gives you piece of mind too. It tells you, look, you’re okay. If they detect something, then they do further tests. But I think they should just leave the PSA alone. (Dale, 70–74, Toronto)

The perception of gender inequity was another issue raised by men in four of the five prostate cancer screening focus groups. They noted that greater attention and public funding are devoted to screening for cancers that are primarily women’s cancers. This perception of gender inequity was particularly an issue in Toronto where “women’s tests” (mammograms and Pap tests) are funded by the provincial health system, whereas the “men’s test” (PSA) is not. While the theme of gender inequity prompted immediate and seemingly affective reactions (feelings of unfairness), it was often underpinned by other more calculative rationales, such as demands for equal access to equivalent services in a publically funded health system.

If it was reversed and government funded the male test but not the female test, wouldn’t that be in the press and you’d have all these protests? (Tony, 55–59, Toronto)

Or, feelings of unfairness were based on economic rationale. One man shared a financial argument as well as the increased risk of cancer as to why it may be unfair for the province of Ontario to not fund PSA testing for asymptomatic men:

If it’s $35.00 it may be enough of a disincentive for someone to say, ‘I’m not going to get it,’ and then you’re a burden on health care because you end up with prostate cancer and getting treatment. (John, 65–69, Toronto)

Men in Toronto were also asked if they thought the provincial government should make opportunistic PSA screening a publicly-funded service. Four of the 16 Toronto participants who provided clear opinions felt the government should fund the PSA test, five felt it should be funded in some cases (e.g., for men with low incomes), while the other seven felt it should not be funded.

Approximately twice as many men who expressed clear opinions about their tolerances for false-positives were fairly comfortable with the risk (13) as those who were not (7). Overall, however, the risk of false-positives was a more frequent topic in the prostate cancer focus groups than in the breast cancer focus groups. This may have been influenced by media coverage of the publication of revised U.S. PSA screening guidelines [76] which highlighted the harms of PSA screening one day before the second of the five prostate cancer screening focus groups. Exposure to this information and the evidence that supported it may have influenced opinions towards a more analytical assessment of risk.

Nevertheless, affective reactions to prospects of false-positives did compel one man who had experienced a false-positive PSA test to state that “It can create a hell of a lot of emotional distress. That’s why I simply decided ‘to hell with it.’ If I have to go [die of cancer], I’m going. I’m not going to go through that [another false-positive]” (Benjamin, 60–64, Toronto). Other men were comfortable with the high risk of false-positives when contextualized within a fear of cancer.

I’d be more perturbed at you giving me the right one [a true-positive test result] because, if it’s a false-positive, it’s false [i.e., no cancer found on further investigation]. I’m going to have a little anxiety, but I ain’t going nowhere [not going to die of prostate cancer]. (Ernest, 65–69, Winnipeg)

I think the PSA is essential. I think screening is good [despite issues of false-positives]. (Dale, 70–74, Toronto)

Some men’s comments often included acknowledgements of the test’s limitations and suggestions that it is only a first step to diagnosing prostate cancer. The following quote displays one man’s attempt to temper the fear and uncertainty that PSA testing can prompt by countering it with a rational calculation of the risk posed by the test.

It’s not that bad. A blood test is not going to say whether you have prostate cancer. All it will say is what your readings are and those can fluctuate. And then if the readings are high, then you go to the next step. (Tony, 55–59, Toronto)

Most men who had positive opinions about PSA screening and acknowledged the test’s unreliability did not explicitly express concerns about screening harms, but men with negative opinions did:

One of the things you read is that people who have had prostate cancer treatment really don’t live a whole lot longer than people that don’t have treatment … [Treatment] outcomes can be very bad. My cousin went through it and he got uncontrollable incontinence. (Howard, 65–69, Winnipeg)

In comparison to the large proportion of men who had generally positive opinions about opportunistic PSA screening, far fewer men supported an organized, population-based PSA screening program: 19 of the 28 men who offered clear opinions opposed organized screening. The other nine men favored expanding PSA screening, but most offered relatively weak opinions (e.g., by simply answering “yes” or “of course” to the question without additional comment). In contrast, most men who opposed expanding screening provided relatively analytical rationales for their opinions, most commonly that resources that would be devoted to a screening program could be better spent on research to develop a better screening test, on cancers with higher mortality rates, or other health problems. Omitting unclear/missing opinions, a larger proportion of men aged 65 to 74 years appeared to support a PSA screening program (4 of 7) than men aged 45 to 64 (5 of 21).

Amongst those who opposed expanding PSA screening (both cities) and/or the funding of the PSA test in Toronto (total *n* = 20), the most common rationale was the test’s unreliability, followed by the cost of a screening program, more urgent health priorities, and that screening recommendations should come from men’s doctors not governments. Among the smaller number of men who supported an organized screening program or (in Toronto) funding the PSA test for screening purposes (total *n* = 11), the most common analytical rationale was the potential savings in treatment costs associated with detecting and treating prostate cancer early. Men aged 65 to 74 were more likely than men aged 45 to 64 to support organized PSA screening and/or (in Toronto) the funding of the PSA test. Amongst men aged 45 to 54 years, only one was in favor, while eight opposed organized screening and funding.

## Discussion

This study adds to the emerging body of research on the influence of affective factors on laypersons’ decision making about cancer screening [29, 30, 51, 52], and affect was indeed a major influence in the cancer screening perspectives of many participants. Participants who had positive opinions about mammography screening (women) or PSA screening (men) expressed enthusiasm almost as a gut reaction for the message that “early detection is your best protection” against cancer, based on affectively-laden expressions of fear towards the disease and empathy towards those who face it. Participants with negative/critical opinions about screening did not voice these kinds of sentiments as often. Affective factors were particularly noticeable in the comments of participants in the sub-groups most supportive of screening: women who felt mammogram screening should begin at age 40 rather than 50, and men who felt that PSA screening should be expanded beyond its current unorganized, opportunistic usage.

Affective factors (i.e. fear or enthusiasm) were also very much intertwined with analytical factors, with each at times providing support and guidance for each other in what Finucane, Slovic, and Peters have dubbed “the dance of affect and reason” [77]. For example, some women who felt strongly positive about starting mammogram screening at 40 years justified their stance by arguing that younger women may have younger children that need their care. One finding that was unique to men was that because of their fear of cancer and of having an undiagnosed and potentially deadly disease without knowing it, many held the belief that the PSA test, despite its unreliability, is “better than nothing” in order to better self-assess their risk. In another instance, men in Ontario argued that men deserve publicly-funded prostate cancer screening because women have publicly-funded breast and cervical cancer screening. Indeed, the latter factor is common in the messages of prostate cancer advocacy organizations [78], but we are not aware of any previous research empirically documenting this gender inequity argument in male patients/laypersons. Nevertheless, negative reactions of unfairness were supported by injustice frameworks, whether in terms of unequal access to care or financial disincentives. Interestingly, these more analytical reactions would find themselves on Siegrist and colleague’s TCC Model within the more intuitively-based Trust domain as judgments on another’s values – where the government is seen as not equally serving the best interests of men compared to women [23–25].

Despite the increasing evidence suggesting the harms of mammography screening in women aged 40 to 49 years may outweigh benefits, and that the harms of PSA screening may outweigh benefits in most men, our findings indicate that some Canadian men and women remain uncertain. Rather than mitigating uncertainty, recent evidence has added greater complexity and confusion created by conflicting and competing messaging from cancer agencies and cancer advocacy organizations. When these conflicting messages enter the public sphere through the news media, this confusion and uncertainty extends to Canadian men and women who are trying to determine what is best for them. Many men and women may have their opinions about cancer screening informed by affective connections about the prospect of cancer – fear of cancer, its potentially early detection and cure, and resultant relief – that may be difficult to reconcile when confronted with studies and recommendation that seem to run counter to those affectively-based beliefs. Beliefs animated in such ways may be difficult to influence, when powerful emotions such as fear and vulnerability are involved. Thus, while affective and analytical domains often function in tandem towards their mutual support, our study sheds more light on the dissonance that can exist between the pathways as well when they seem to come into conflict. To this end, our study offers further support to literature that holds that when faced with an increasingly complex and uncertain situation, reflexively giving primacy to their affective impressions about testing or screening for cancer may help people to cope with and orient themselves to an increasingly uncertain situation with seemingly conflicting information [10, 30].

Considerable uncertainty remains at the policy level as well, which is reflected in the lack of changes in Canada’s provincial/territorial mammography screening programs since the 2011 publication of the Canadian Task Force on Preventive Health Care’s revised recommendation for women aged 40 to 49 years. Similarly, opportunistic PSA screening in Canada remains widespread (and publicly funded in most Canadian provinces) despite evidence about minimal benefits and considerable harms. In light of the results here, the lack of changes can likely be associated with the continued resonance and positive affective associations that “early detection” messaging holds in the minds of the public, which can be held alongside the fear that people have towards cancer. With powerful advocacy groups employing strong affective messaging that keep such mantras reflexively at hand, for policy experts that wish to balance support for screening with emerging evidence, some degrees of policy deadlock could be expected.

It is not entirely surprising that mammography screening policy and practice has not shifted dramatically since the CTFPHC’s 2011 recommendation that women aged 40 to 49 should not be screened. Unlike the CTFPHC expert panel’s members who have full independence from government, civil servants at cancer agencies and ministries of health are sensitive to and affected by the political process and the platforms of the governments they serve. Given the emotionally charged nature of cancer, maintaining the status quo may be an attractive default policy response, even if the evidence suggests a different alternative.

Moreover, the CTFPHC recommendations are primarily based on a specific form of evidence: clinical data from randomized controlled trials. Alternate forms of evidence, often deemed of lower quality, are given less consideration: e.g., observational studies and other non-randomized clinical study designs, as well as research about patient preferences, ethical concerns over the use of scarce resources, and economic evaluations [79]. Nevertheless, these other forms of evidence are frequently found in the arguments of cancer advocacy organizations and professional organizations (e.g. urology, radiology) when they challenge CTFPHC recommendations. These other forms of evidence are also often present in the analytical and affective language used by members of the public and some of this study’s focus group participants: e.g., gender inequities over screening tests for men compared to women; being more convinced that the perceived certainty of saving one life is preferable to the risk of harms large numbers of people may experience as a result of screening; or that the lack of funding for a test can place lives needlessly at risk by seeming to prioritize reducing costs over saving lives.

From a health communications perspective, our findings offer a mix of good news and bad news for policymakers and physicians. There was a broad diversity of opinions about the overall value of mammography and PSA screening. In general, women in the breast cancer screening focus groups were more supportive of organized mammography screening than men were towards prostate cancer screening. Most men supported the opportunistic screening that is occurring through widespread clinical use of the PSA test, but did not support the development of an organized screening program, primarily because of the test’s unreliability.

The good news for policymakers and physicians is that the discussion of screening harms appears to be comparatively effective with the age groups at lower risk of breast or prostate cancer. A recent Australian study about women’s perceptions of overdiagnosis and overtreatment resulting from mammography screening also found that younger women were more concerned about these harms [80]. Likewise, our study’s younger participants, especially women, were more concerned about the relative benefits and harms of screening than older participants. The potential for false-positive results was a more frequent topic of discussion in the prostate cancer focus groups, but most men who expressed an opinion were comfortable with the risk of false-positive PSA test results, whereas most women who provided an opinion said they were *not* comfortable with the risk of false-positive mammogram results. (A particular cohort effect relevant to the timing of the men’s focus groups may have played a role in influencing some of their perspectives of harms associated with PSA testing – see Methodological Issues and Limitations section below).

Older participants (women 50 years and older, men 65 years and older) had greater affective attachments to fear and anxiety about cancer as well as a more generalized enthusiasm towards the message that early detection is the best protection against cancer. Past experience (the analytical domain of Siegrist and colleagues’ conceptual model [23]) may play a role here too, as older participants ‘experienced’ stories of cancer as “the Big C” during an earlier time when advancements in treatments and overall survival were poorer, and so existing tests could truly be seen as “better than nothing.” Moreover, they would have had more cumulative exposure to “early detection” messages, as well as more experience having mammograms and PSA tests, thus being more comfortable with them as routine services even if they held the potential for false positives. Finally, given the age-based risk associated with the cancers in question, they would also be more likely to know someone with a respective cancer (along with the accumulated affective imprints those anecdotes can produce). In the same vein as Slovic [10] and Farrell, Murphy, and Schneider [30] have argued, their fear of cancer and positive feelings toward the prospect of testing could move them to judge the risks as low and the benefits as high.

The bad news for policymakers and physicians is that Canadian cancer advocacy organizations appear to be aware that younger men and women have less enthusiastic attachments to the message of early detection. These organizations are aggressively promoting breast and prostate cancer awareness and screening to younger women and men with campaigns that compellingly mix selected evidence with affect-inducing language and imagery [34–38, 67].

Interestingly, however, affective language is also being used in messaging about screening harms. In an article on the web site of Time magazine, a table illustrating the benefits and harms of mammography referred to false-positive results as “false diagnoses,” and added the affectively-charged (anxiety-inducing) phrase that these are “often associated with months of waiting for all-clear.” Similarly, the overtreatment category of harm was accompanied by a footnote specifying that overtreatment is “complete or partial breast removal” [81].

That said, people on all sides of the issue marshal specific evidence to support their positions, each imparting the notion that a correct answer exists. This is both a strength and a limitation of an evidence-informed approach: the subjective definition of *correct* depends on the outcomes selected, and whether or not individual values, preferences and costs are subordinate to or trump clinical evidence.

Of course, another factor that may circumvent patient preferences and attitudes concerns the dynamics of the clinician-patient encounter itself. It is not only issues such as patient perceptions of risk or their affective states that may prove an obstacle to changing policy or practice. Physicians often resist change in their current practices and routines, even if they run counter to emerging evidence [82]. For example, many physicians routinely order PSA tests during unrelated physical examinations, and patients may not even know that it is being done [83]. Although considered ideal practice, studies have shown a considerable lack of shared decision making between patients and clinicians regarding cancer screening decision-making [84], and there are often inadequate discussion of harms and benefits [85]. At the same time, patient trust and willingness to comply with their doctor’s recommendation is yet another influential context that can involve intuitive (‘gut reactions’ of whether their doctor ultimately has their best interest in mind) and analytical (based on past experience with their doctor) influencers on screening behavior.

### Policy and practice implications

We found that younger women and men are more likely to be influenced by evidence about the harms they may experience from mammogram/PSA screening, but older family members or friends who have affective attachments to the “early detection” message may influence the screening decisions of younger persons. Policymakers and physicians can anticipate and counter that possibility with messages reinforcing why women aged 50 to 69 are included in mammogram screening programs, why women under 50 are not screened, and why men may want to think carefully about PSA screening. In addition to evidence about the benefits and harms of screening, these messages should also incorporate affective content about harms because false-positives, unnecessary biopsies, overdiagnosis, and overtreatment, in and of themselves, these words do not intrinsically arouse emotional reactions as immediately and dramatically as death from cancer.

### Methodological issues and limitations

Because this study only sampled in two cities, our findings cannot be generalized to the population of all men and women in Canada. Nevertheless, our focus group participants had a diverse mix of socio-demographic characteristics, so our findings suggest trends that may be indicative of the population. Educational attainment was skewed to higher levels, particularly among women, so our findings may not be representative of women without at least some post-secondary education.

For the breast cancer screening focus groups, both cities were in provinces where routine mammogram screening begins at age 50, so our findings may have been different if focus groups had also been conducted in a province that begins screening women at age 40. The United States Preventive Services Task Force published a revised recommendation against PSA screening [76] one day before the second of the five prostate cancer screening focus groups, and it received widespread Canadian media coverage [86, 87]. This likely heightened awareness of the limitations of PSA screening in men who attended the focus groups.

Precise quantification of results is not a fundamental component of qualitative research, but some quantification provides an indication of the absolute and relative occurrence of particular findings. Where quantitative findings have been reported, there are sometimes large proportions of “unclear” responses (i.e., where analysis could not establish a clear answer for some participants) because focus group discussions are not suited to obtaining precise answers to every question from every participant.

## Conclusion

Consideration of affective influences on laypersons’ screening decisions provides a more comprehensive way of understanding the complex interplay of factors that are involved; even when these decisions appear to not be in their best interests and/or at odds with evidence and clinical guidelines. To the question of whether you can “un-ring the bell” of “early detection” messaging for some cancers, the answer appears to be a mixture of “yes” and “no”. Younger men and women in our focus groups tended to be more receptive to evidence about the harms of screening, while older participants were more attached to the affect inducing “early detection” message that has guided cancer screening programs for decades. Nevertheless, affect played a role in the screening decisions of many participants, younger and older, so policymakers and physicians attempting to communicate more nuanced versions of the “early detection” message need to understand the role of affect in laypersons’ decision making, and anticipate how affective responses to their messages will be shaped, transformed, and potentially subverted by external forces beyond their control (e.g., media coverage, cancer advocacy groups, specialist organizations).

## Additional files

Additional File 1: Detailed Methods for “Can you un-ring the bell? A qualitative study of how affect influences cancer screening decisions”. This document provides a much more detailed of the study’s methods, with particular close attention to the process of data collection. (DOCX 35 kb)

Additional File 2: Mammography Screening Focus Group Interview Guide. This document contains the semi-structured focus group instrument that guided discussions with women about mammography screening. The document also contains a graphic that was handed out to participants at the mid-way point of the focus groups that illustrates the number of women needed to be tested to prevent one death. Source of graphic was the Canadian Task Force on Preventive Health Care. “Recommendations on screening for breast cancer in average-risk women aged 40-74 years.” Canadian Medical Association Journal, November 22, 2011. (PDF 198 kb)

Additional File 3: Focus Group Guide – Prostate Cancer Screening. This document contains the semi-structured focus group instrument that guided discussions with men about prostate cancer screening. The document also contains a handout that was distributed to participants at the mid-way point of the focus groups with summarizes the current research about prostate cancer screening. (PDF 149 kb)

## Abbreviations

CTFPHC: Canadian Task Force on Preventive Health Care
PSA: Prostate-specific antigen
USPSTF: United States Preventive Services Task Force

## References

[1] The Canadian Task Force on Preventive Health Care (2011). Recommendations on screening for breast cancer in average-risk women aged 40–74 years. Can Med Assoc J.

[2] Bell N, Connor Gorber S, Shane A, Joffres M, Singh H, Dickinson J, et al. Recommendations on screening for prostate cancer with the prostate-specific antigen test. Can Med Assoc J. 2014; doi: 10.1503/cmaj.140703. 10.1503/cmaj.140703 PMC4216256 25349003

[3] Esserman L, Shieh Y, Thompson I (2009). Rethinking screening for breast cancer and prostate cancer. JAMA.

[4] Chaiken S (1980). Heuristic versus systematic information processing and the use of source versus message cues in persuasion. J Pers Soc Psychol.

[5] Petty RE, Cacioppo JT (1986). The elaboration likelihood model of persuasion. Adv Exp Soc Psychol.

[6] Epstein S (1994). Integration of the cognitive and the psychodynamic unconscious. Am Psychol.

[7] Chaiken S, Trope Y (1999). Dual-process theories of social psychology.

[8] Cameron LD, Leventhal H (2003). The self-regulation of health and illness behavior.

[9] Dunwoody S, Griffin RJ, Arvai J, Rivers L (2014). The role of channel beliefs in risk information seeking. Effective risk communication.

[10] Slovic P, Peters E, Finucane ML, MacGregor DG (2005). Affect, risk, and decision making. Health Psychol.

[11] Zajonc R (1980). Feeling and thinking: preferences need no inferences. Am Psychol.

[12] Johnson E, Tversky A (1983). Affect, generalization, and the perception of risk. J Pers Soc Psychol.

[13] Schwarz N, Clore G, Fiedler K, Forgas J (1988). How do I feel about it? Informative functions of affective states. Affect, Cognition, and Social Behavior.

[14] Lowenstein G, Weber E, Hsee C, Welch N (2001). Risk as feelings. Psychol Bull.

[15] Finucane M, Alhakami A, Slovic P, Johnson S (2000). The affect heuristic in judgments of risks and benefits. J Behav Dec Making.

[16] Slovic P, Finucane ML, Peters E, MacGregor DG (2004). Risk as analysis and risk as feelings: some thoughts about affect, reason, risk, and rationality. Risk Anal.

[17] McAllister D (1995). Affect- and cognition-based trust as foundations for interpersonal cooperation in organizations. Acad Manag J.

[18] Tversky A, Kahneman D (1974). Judgment under uncertainty: heuristics and biases. Science.

[19] Tversky A, Kahneman D (1973). Availability: a heuristic for judging frequency and probability. Cogn Psychol.

[20] Wilson RS, Arvai JL (2010). Why less is more: exploring affect-based value neglect. J Risk Res.

[21] Wilson RS, Arvai JL (2006). When less is more: how affect influences preferences when comparing low and high-risk options. J Risk Res.

[22] Slovic P, Finucane ML, Peters E, MacGregor DG, Gilovich T, Griffin D, Kahneman D (2002). The affect heuristic. Heuristics and biases: the psychology of intuitive Judgement.

[23] Siegrist M, Earle T, Gutscher H (2003). Test of a trust and confidence model in the applied context of electromagnetic field (EMF) risks. Risk Anal.

[24] Siegrist M, Gutscher H, Earle T (2005). Perception of risk: The influence of general trust, and general confidence. J Risk Res.

[25] Earle T, Siegrist M (2008). Trust, confidence and cooperation model: a framework for understanding the relation between trust and risk perception. Int J Global Environ Issues.

[26] Clarke JN, Everest MM (2006). Cancer in the mass print media: fear, uncertainty and the medical model. Soc Sci Med.

[27] Rimer BK, Briss PA, Zeller PK, Chan ECY, Woolf SH (2004). Informed decision making: what is its role in cancer screening?. Cancer.

[28] Lee SJC (2010). Uncertain futures: individual risk and social context in decision-making in cancer screening. Health Risk Soc.

[29] Kiviniemi MT, Jandorf L, Erwin DO (2014). Disgusted, embarrassed, annoyed: affective associations relate to uptake of colonoscopy screening. Ann Behav Med.

[30] Farrell M, Murphy M, Schneider C (2002). How underlying patient beliefs can affect physician-patient communication about prostate-specific antigen testing. Eff Clin Pract.

[31] Visschers VHM, Wiedemann PM, Gutscher H, Kurzenhäuser S, Seidl R, Jardine CG (2011). Affect-inducing risk communication: current knowledge and future directions. J Risk Res.

[32] Woloshin S, Schwartz LM (2012). How a charity oversells mammography. BMJ.

[33] Gigerenzer G (2014). Breast cancer screening pamphlets mislead women. BMJ.

[34] Team Shan. Team Shan: Breast cancer awareness for young women. http://www.teamshan.ca. Accessed 31 Aug 2017.

[35] Canadian Breast Cancer Foundation. My Breast My Test (Facebook). https://www.facebook.com/MyBreastMyTest. Accessed 31 Aug 2017.

[36] Canadian Breast Cancer Foundation. The facts: Breast cancer in young women. Canadian Breast Cancer Foundation. http://www.cbcf.org/central/YourDollarAtWork/Grants/Pages/BCYW2014Facts.aspx. Accessed 31 Aug 2017.

[37] Rethink Breast Cancer. http://rethinkbreastcancer.com/. Accessed 31 Aug 2017.

[38] Support PSA Tests. Prostate Cancer Canada. http://www.supportpsatests.ca. Accessed 31 Aug 2017.

[39] Domenighetti G, D'Avanzo B, Egger M, Berrino F, Perneger T, Mosconi P (2003). Women's perception of the benefits of mammography screening: population-based survey in four countries. Int J Epidemiol.

[40] Hudson B, Zarifeh A, Young L, Wells JE (2012). Patients’ expectations of screening and preventive treatments. Ann Fam Med.

[41] Gigerenzer G, Mata J, Frank R (2009). Public knowledge of benefits of breast and prostate cancer screening in Europe. J Natl Cancer Inst.

[42] Dickinson J, Shane A, Tonelli M, Gorber SC, Joffres M, Singh H (2016). Trends in prostate cancer incidence and mortality in Canada during the era of prostate-specific antigen screening. CMAJ Open.

[43] Welch HG, Black WC (2010). Overdiagnosis in cancer. J Natl Cancer Inst.

[44] Gotzsche PC, Jorgensen KJ, Maehlen J, Zahl PH (2009). Estimation of lead time and overdiagnosis in breast cancer screening. Br J Cancer.

[45] Duffy SW, Lynge E, Jonsson H, Ayyaz S, Olsen AH (2008). Complexities in the estimation of overdiagnosis in breast cancer screening. Br J Cancer.

[46] Iremashvili V, Pelaez L, Manoharan M, Jorda M, Rosenberg DL, Soloway MS (2012). Pathologic prostate cancer characteristics in patients eligible for active surveillance: a head-to-head comparison of contemporary protocols. Eur Urol.

[47] Consedine NS, Magai C, Krivoshekova YS, Ryzewicz L, Neugut AI (2004). Fear, anxiety, worry, and breast cancer screening behavior: a critical review. Cancer Epidemiol Biomark Prev.

[48] Bowen DJ, Helmes A, Powers D, Andersen MR, Burke W, McTiernan A (2003). Predicting breast cancer screening intentions and behavior with emotion and cognition. J Soc Clin Psychol.

[49] Diefenbach MA, Miller SM, Daly MB (1999). Specific worry about breast cancer predicts mammography use in women at risk for breast and ovarian cancer. Health Psychol.

[50] Hay JL, Buckley TR, Ostroff JS (2005). The role of cancer worry in cancer screening: a theoretical and empirical review of the literature. Psychooncology.

[51] Kiviniemi MT, Hay JL, James AS, Lipkus IM, Meissner HI, Stefanek M (2009). Decision making about cancer screening: an assessment of the state of the science and a suggested research agenda from the ASPO behavioral oncology and cancer communication special interest group. Cancer Epidemiol Biomark Prev.

[52] Myers RE (2005). Decision counseling in cancer prevention and control. Health Psychol.

[53] Wegwarth O, Gigerenzer G (2013). Less is more: Overdiagnosis and overtreatment: evaluation of what physicians tell their patients about screening harms. JAMA Intern Med.

[54] Schwartz LM, Woloshin S, Sox HC, Fischhoff B, Welch HG (2000). US women's attitudes to false positive mammography results and detection of ductal carcinoma in situ: cross sectional survey. BMJ.

[55] Morrison BJ (1994). Screening for breast cancer. The Canadian guide to clinical preventive health care.

[56] Ringash J (2001). The Canadian task force on preventive health care. Preventive health care, 2001 update: screening mammography among women aged 40–49 years at average risk of breast cancer. Can Med Assoc J.

[57] Weeks C. New breast cancer screening guidelines inflame debate, add to confusion. Toronto: Globe and Mail. November 21, 2011.

[58] Canadian Partnership Against Cancer (2013). Organized breast cancer screening programs in Canada: report on program performance in 2007 and 2008.

[59] Canadian Partnership Against Cancer (2014). Breast cancer screening guidelines across Canada: environmental scan.

[60] Canadian Partnership Against Cancer (2012). Breast control in Canada: a system performance focus report.

[61] Rossi R. Scrapping the PSA test for prostate cancer is an injustice to men. Toronto: Globe and Mail. October 28, 2014.

[62] Canadian Urological Association. Press release to: Canadian urological community. October 27, 2014.

[63] Prostate Cancer Canada. Prostate Cancer Canada reminds men that early detection using ‘smart screening’ for prostate cancer can save lives: Prostate Cancer Canada; 2014.

[64] Ubelacker S. Canadian task force advises against screening for prostate cancer using PSA test. Winnipeg: Winnipeg Free Press. October 27, 2014.

[65] Kirkey S. Expert panel urges Canadian doctors to stop screening for prostate cancer. Toronto: Postmedia News. October 27, 2014.

[66] Izawa J, Klotz L, Siemens DR, Kassouf W, So A, Jordan J (2011). Prostate cancer screening: Canadian guidelines 2011. Can Urol Assoc J.

[67] Prostate Cancer Canada (2013). PSA recommendation: know your number.

[68] Canadian Cancer Society's Advisory Committee on Cancer Statistics (2012). Canadian cancer statistics 2012.

[69] Beaulac JA, Fry RN, Onysko J (2006). Lifetime and recent prostate specific antigen (PSA) screening of men for prostate cancer in Canada. Can J Public Health.

[70] Sher J. Prostate cancer screening test costs Ontario patients $30. Toronto: Toronto Star. May 21, 2012.

[71] Krueger RA (1988). Focus groups: a practical guide for applied research.

[72] Morgan DL, Krueger RA, Morgan DL (1993). When to use focus groups and why. Successful focus groups: advancing the state of the art.

[73] Liamputtong P (2011). Focus group methodology: principles and practice.

[74] Neuendorf KA (2002). The content analysis guidebook.

[75] Strauss AL, Corbin J (1998). Basics of qualitative research: techniques and procedures for developing grounded theory.

[76] Moyer VA (2012). Screening for prostate cancer: U.S. preventive services task force recommendation statement. Ann Intern Med.

[77] Finucane M, Peters E, Slovic P, Schneider S, Shanteau J (2003). Judgment and decision making: the dance of affect and reason. Emerging perspectives on judgment and decision research.

[78] Rossi R. Ontario should pay for prostate cancer testing. Toronto: Toronto Star. May 28, 2014.

[79] Krahn M (2014). Prostate cancer screening: going beyond the clinical evidence. CMAJ.

[80] Hersch J, Jansen J, Barratt A, Irwig L, Houssami N, Howard K (2013). Women's views on overdiagnosis in breast cancer screening: a qualitative study. BMJ.

[81] Gigerenzer G. This one graphic will change the way you look at breast cancer screening. Time. 2014; http://time.com/83305/breast-cancer-mammogram-statistics-mortality. Accessed 31 Aug 2017

[82] Cabana M, Rand C, Powe N, Wu A, Wilson M, Abboud P (1999). Why don't physicians follow clinical practice guidelines? A framework for improvement. JAMA.

[83] Sirovich BE, Schwartz LM, Woloshin S (2003). Screening men for prostate and colorectal cancer in the united states: does practice reflect the evidence?. JAMA.

[84] Hoffman RM, Elmore JG, Fairfield KM, Gerstein BS, Levin CA, Pignone MP (2014). Lack of shared decision making in cancer screening discussions: results from a national survey. Am J Prev Med.

[85] Hoffman RM, Lewis CL, Pignone MP, Couper MP, Barry MJ, Elmore JG (2010). Decision-making processes for breast, colorectal, and prostate cancer screening: the DECISIONS survey. Med Decis Mak.

[86] CBC News (2012). Routine PSA prostate cancer tests not recommended.

[87] Picard A. Warning on prostate tests sparks debate. Toronto: Globe & Mail. May 22, 2012; p. A1.

