# Supplementary material for: Can you un-ring the bell? A qualitative study of how affect influences cancer screening decisions
Source: BMC Cancer. 2017 Sep 13;17:647. doi: 10.1186/s12885-017-3596-7 (PMC5598010; doi:10.1186/s12885-017-3596-7)
Supplement: Supplementary file 1 — Detailed Methods for “Can you un-ring the bell? A qualitative study of how affect influences cancer screening decisions”. This document provides a much more detailed of the study’s methods, with particular close attention to the process of data collection. (DOCX 35 kb) [file 12885_2017_3596_MOESM1_ESM.docx]

**Additional File 1: Detailed Methods for “Can you un-ring the bell? A qualitative study of how affect influences cancer screening decisions”**

## Study Overview

This research was conducted as part of a broad mixed-methods study examining the management of uncertainty in decision making about cancer control policy in Canada that is funded by a grant held by author SMD from the Canadian Cancer Society Research Institute entitled “Advancing quality in cancer control and cancer system performance in the face of uncertainty” (grant #700589). Authors SMD and MB are the co-principal investigators. The study is examining four specific cancer control scenarios that are characterized by varying kinds and levels of uncertainty: (1) mammography screening in women aged 40 to 49 years, (2) prostate-specific antigen (PSA) screening, (3) the approval and funding of new cancer drugs, and (4) school-based HPV vaccination programs.

The study has three broad components: (1) an integrated review of empirical research (quantitative and qualitative) and non-empirical works (e.g., theory, conceptual frameworks, commentary, etc.) about uncertainty in health policy decision making; (2) qualitative research involving focus groups with members of the public and key informant interviews with senior officials who make decisions about or influence policy in various organizations in the Canadian cancer control system; and (3) quantitative empirical research leading to the development of a practical tool to help policymakers assess and manage uncertainty in their analyses, and ultimately make better and more transparent decisions about cancer control policy. This paper deals exclusively with component (2).

## Ethics

The research protocol, consent forms, and data collection instruments received ethics approval from the University of Manitoba’s Health Research Ethics Board (H2010:194).

## Participants and Recruitment (Mammography and PSA Screening Scenarios)

Women aged 35 to 59 years (n = 46) and men aged 45 to 74 (n = 47) were recruited for focus groups about, respectively, breast and prostate cancer screening. Different age ranges were recruited for the two scenarios because of the epidemiological differences of the two cancers (breast cancer is more common than prostate cancer under the age of 50, whereas most prostate cancers occur in men over 60) [[1](#_ENREF_1)]. Two survey research companies (Nexus Market Research in Toronto, Prairie Research Associates in Winnipeg) recruited the focus group participants by telephone using combinations of existing databases of persons who had agreed to be contacted about surveys, and random digit dialing. The companies screened potential participants for eligibility using a questionnaire developed by the research team. Preliminary screening questions excluded persons outside of the eligible age groups, persons with a history of cancer, and health care workers. Using maximum variation sampling [[2](#_ENREF_2)], persons who expressed interest in attending a focus group were asked a series of socio-demographic questions to ensure each focus group included participants with a diverse mix of characteristics known to influence public perceptions of risk (e.g. income, education levels) [[3](#_ENREF_3)].

## Data Collection

Established protocols for conducting focus groups were followed [[4-6](#_ENREF_4)]. All participants gave informed consent prior to participation and publication of results. Ten focus groups about breast cancer (n = 5) and prostate cancer (n = 5) screening were held in Toronto, Ontario (n = 6) and Winnipeg, Manitoba (n = 4) in May and June of 2012. This was shortly after the publication of new mammography and PSA screening recommendations in, respectively, Canada and the U.S. The Canadian Task Force on Preventive Health Care published its current breast cancer screening guidelines in November 2011 [[7](#_ENREF_7)], and the United States Preventive Services Task Force published revised guidelines recommending against the use of the PSA test to screen for prostate cancer [[8](#_ENREF_8)] between the first and second prostate cancer groups. The first group in each screening scenario was a pilot test of the interview guide.

| **Cancer Screening Focus Group Schedule** | | | | |
| --- | --- | --- | --- | --- |
| Breast Cancer (women) | |  |  |  |
| Date | Location | Age group | # of participants | Notes |
| May 16, 2012 | Toronto | 35-59 | 8 | pilot |
| May 22, 2012 | Toronto | 35-49 | 9 |  |
| May 24, 2012 | Toronto | 45-59 | 10 |  |
| May 28, 2012 | Winnipeg | 35-49 | 9 |  |
| May 29, 2012 | Winnipeg | 45-59 | 10 |  |
| Total women |  |  | 46 |  |
|  |  |  |  |  |
| Prostate Cancer (men) | |  |  |  |
| May 17, 2012 | Toronto | 45-74 | 10 | pilot |
| May 23, 2012 | Toronto | 45-59 | 7 |  |
| May 24, 2012 | Toronto | 60-74 | 10 |  |
| June 4, 2012 | Winnipeg | 45-59 | 11 |  |
| June 5, 2012 | Winnipeg | 60-74 | 9 |  |
| Total men |  |  | 47 |  |

To assess the impact of age on participants’ responses, eight of the ten focus groups were stratified by age. For prostate cancer screening, two focus groups were restricted to men aged 45 to 59 years and two were restricted to men aged 60 to 74 years. For breast cancer screening, two focus groups were restricted to women aged 35 to 49 and two were restricted to women aged 45 to 59. Both of these sub-groups included women aged 45 to 49 years to assess the perspectives of women in that age group when in the presence of younger women (35-44 years) compared to when they were with older women (50-59 years). The focus group interview guides were pilot tested in two focus groups (one prostate cancer, one breast cancer). The final version of the instruments did not include any substantial revisions, so the data from the pilot focus groups have been pooled with the remaining focus groups for analysis.

The focus group discussions were guided by an interview guide that began with open-ended questions about cancer, tests for detecting cancer, participants’ understandings of population-based screening, and how they made decisions about their health. At the mid-point of each meeting, the interviewer gave plain language descriptions of current guidelines for breast/prostate cancer screening, a summary of the research evidence upon which those guidelines were based, and a description of the uncertainties that remained. These presentations were accompanied by print hand-outs given to each participant. Following these presentations, women were asked at what age they thought mammogram screening should begin in their province, and men were asked if they thought there should be an organized prostate cancer screening program in their province. Men in Toronto were also asked if they thought the Ontario provincial healthcare system should cover the cost of PSA tests for screening. Each focus group participant received a $60 honorarium.

## Data Analysis

All focus groups were digitally audio-recorded, transcribed verbatim, audio-verified against the recordings and edited to correct any errors. All data were analyzed using NVivo10^TM^ qualitative data management software. The project lead, SMD, is an expert in qualitative methods and a trainer for using NVivo ^TM^. Her typical strategy for coding data involves doing a first level of coding of surface level descriptive content. What this entails is to code lines of text into different content categories of meaning in order to group those ‘like’ ideas within the same category across the dataset. For example, participants were asked about what risks or harms had ever been mentioned to them or that they read if they sought out information about mammography screening/PSA testing. Any reference that participants raised about risks would be coded in this node. This enables the researcher to examine how people discuss and contextualize notions of ‘risk’ however defined by the participants.

Because qualitative analysis does not aim to precisely quantify qualitative data (e.g. X% of lines of text across the dataset focused on Idea Y), it is common, and in fact strongly recommended, to code lines of text multiple times into relevant content categories as appropriate. For example, Bazeley [[9](#_ENREF_9), [10](#_ENREF_10)] recommends using fewer descriptive content categories or nodes, but capturing relevant aspects in terms of what is being described, e.g. is it being described positively or negatively (if that can be assessed or relevant), and so forth. The aim is to be able to put together more sophisticated questions that can be asked of the dataset through queries once the dataset has been initially coded.

SMD operationalizes this strategy by dividing surface descriptive content categories very broadly into *who, what, why*, and occasionally *where and when* categories as relevant. A who-type-category is designed to capture the people or organization being discussed (e.g. health care provider, family/friends, government, media, experts, celebrities, etc). A what-type-category involves the subject matter of what is being described in the sentence, sentence fragment, paragraph, or conversation between a set of participants (as in the case of focus groups). A why-type-category captures more interpretive aspects within any set of text. For example, if a participant describes that they have a family history of breast cancer, and goes into why she felt she had to be pushier with her doctor to be screened before she was 50 would be coded as follows: decision-making process (because family history was her expressed motivation to become pushy), family (to capture her references to her family history), physician (because she is discussing strategies she used with her doctor), risk group (because she feels she might be at higher risk for breast cancer because of her family history), and so forth depending on the exact nature of the lines of text in question. Likewise, if someone is talking about something in a negative or positive manner, these also become coded as positive or negative. These nodes are more liberally assigned and, occasionally subjectively interpreted by the individual coder, which is why processes of reflexivity and researcher triangulation (described further below) are needed. In building on the previous example above, instances of a participant describing her concerns about her history with her physician but not feeling that her physician is being sufficiently attentive to her worries, could see this coded as ‘negative’. Likewise, someone under the same condition, if finding their physician is quickly receptive to their concerns, might become coded as positive.

Following this general process that structures very broadly who, what and why type categories, authors SMD, GA and two other research staff developed a codebook by independently reviewing a sample of transcript excerpts and iteratively developing draft codes and operational definitions based on the descriptive content of the text itself. After comparing their draft coding schemes, they resolved disagreements through consensus, and eventually agreed on the final coding framework that was used to systematically code each line of transcript text into content categories. As multiple coders were required for this and a related study, four coders would test-coded a sample of transcript excerpts to establish inter-coder reliability. After they coded each transcript excerpt, NVivo’s coding comparison query was used to output Cohen’s Kappa coefficients comparing each pair of coders’ agreements and disagreements for each node. The individual Kappa scores for each node were summed and averaged in an Excel spreadsheet. The coders met to review their Kappa scores, examined the different ways each had coded nodes with particularly low Kappa scores, and collaboratively developed more precise operational definitions to be applied in test-coding the next sample transcript. Kappa scores increased as each subsequent transcript was test-coded, until coders achieved Kappa scores of 0.91, exceeding the common > 0.80 benchmark for near perfect inter-coder reliability [[11](#_ENREF_11)]. RM and three other coders subsequently coded all the data. Salient categories emerging from the surface content coding of data and included: decision-making factors, emotions, risk/risk groups, finance/monetary issues, age, family/friends, side effects/harms, uncertainty, PSA, mammograms, and government policy.

In addition to this, transcripts, when they were being audio-verified, were also verified as best as possible for voice-attribution (i.e. to identify each speaker). Having transcripts organized by voice attribution allows for a number of different ways the data can be analyzed. First, it enables examining how an individual expressed themselves throughout the entire focus group. In a software like NVivo, it is possible to autocode all individual speakers of a focus group into a case-node. Effectively, this creates a separate node for each individual speaker where everything s/he says is coded as their name. This is important because the researcher can subsequently examine how a specific participant discussed the topic throughout the focus group, as well as assess how s/he changed responses over the course of the focus group discussion. While it is never possible to fully ‘know’ why a person changes their perspective within a focus group, training, experience, and careful reading of the text, both as an individual case node, as well as through a participant’s conversation interactions with others, can help to ascertain if someone shifted their response given the presence of a dominant speaker or an emerging ‘group think’, or if they seemed to be changing their response because they were being exposed to information that was new to them, prompting them to rethink their original position.

Second, in doing voice-attribution within a focus group dataset, it is then possible to attach specific attribute level data to each case node in the Node Classifications function of the software. Attribute level data consists of organizational or administrative data information (e.g. age, gender, income, etc), but the researcher can also assign attribute level data to a participant based on how s/he discussed aspects in the focus group. It is important to note that attribute level data is categorical data, not interpretive data, and needs to apply to the whole case (in this case the whole participant). Researcher created attribute level data would include aspects like: participant expressed opinion on age at which mammography screening should begin (i.e. where listed options would include at 40, at 50, <40, unassigned); whether PSA screening should be expanded beyond its opportunistic pattern (i.e. yes, no); and so forth. Attribute level data in an NVivo project, when set up properly, allows for more detailed queries to be run of how participants discussed an issue that was then coded as an interpretive node category against specific classifications/attributes for specific sets of participants. For example, a query of how women discussed their perceived values or benefits from cancer screening programs against attribute level information that screening should begin at age 40. These types of queries allow the researcher to isolate those participants within a larger dataset holding particular views. Moreover, the researcher, in reviewing a series of single query outputs, can conduct a deeper reading of that text to assess the meaning participants were aiming to express again other factors (e.g. outputs from other queries, how issues have been discussed in the literature, etc).

Fundamentally embedded into the surface descriptive coding process as described above, as well as involved with more interpretive reading of the text described further below, is how a software like NVivo allows the researcher to explore ideas through the development of different Memos throughout the coding process, following protocols suggested by Richards [[12](#_ENREF_12)]. As a software tool, all data imported into the project and created within the project is stored together in a single file. Developing Memos, which effectively have the same functionality as a word-processing document, allows the researcher to explore ideas as they emerge. When doing surface level descriptive content coding, a researcher needs to frequently interrupt coding to capture elements that strike them as ‘interesting’ to examine what it is about those lines of text that captured their interest. As more interpretive deep reading occurs as a person conducts different queries of their data, Memos are to be built upon and expanded by incorporating outside literature as well as how different aspects seem to be challenged by some participants, as an example.

Consequently, research findings as reported in the associated article that assign interpretive labels that indicate a participant was discussing cancer screening more affectively or analytically was never specifically coded that way at the outset. Rather, it emerges from a deeper reading of more surface descriptive coding, alongside other coding queries. As the team processes the content of the qualitative data analysis, they assess what results cohere for different papers to be developed in reporting the data. In developing any manuscript, much more detailed and interpretive analyses are undertaken. It is through processes of reflexivity and researcher triangulation [[2](#_ENREF_2)]– where research findings and emerging analysis are discussed with other team members having different disciplinary perspectives – that any emerging analysis is challenged to assess if alternative interpretations are possible. These efforts are imperative to ensure that there is not premature closure in the analysis process. Moreover, the process of constant-comparison coding also serves to challenge the analysts in their thinking to assess if there are other interpretations possible within the text.

**References**

1. Canadian Cancer Society's Advisory Committee on Cancer Statistics. Canadian Cancer Statistics 2014. Toronto: Canadian Cancer Society; 2014.

2. Patton MQ. Qualitative Research & Evaluation Methods. 3rd ed. Thousand Oaks: Sage Publications; 2002.

3. Slovic P. The Perception of Risk. Sterling: Earthscan Publishing; 2000.

4. Krueger RA. Focus groups: A practical guide for applied research. Newbury Park: Sage Publications; 1988.

5. Morgan DL, Krueger RA. When to Use Focus Groups and Why. In: Morgan DL, editor. Successful Focus Groups: Advancing the State of the Art. Newbury Park, CA: Sage Publications; 1993. p. 3-19.

6. Liamputtong P. Focus Group Methodology: Principles and Practice. Los Angeles: Sage Publications; 2011.

7. The Canadian Task Force on Preventive Health Care. Recommendations on screening for breast cancer in average-risk women aged 40–74 years. Can Med Assoc J. 2011;183(17):1991-2001.

8. Moyer VA. Screening for Prostate Cancer: U.S. Preventive Services Task Force Recommendation Statement. Ann Intern Med. 2012;157(2):120-34.

9. Bazeley P. Qualitative Data Analysis with NVivo. London: Sage Publications; 2007.

10. Bazeley P, Jackson K. Qualitative data analysis with NVivo. 2nd ed. Los Angeles: Sage Publications; 2013.

11. Neuendorf KA. The Content Analysis Guidebook. Thousand Oaks: Sage; 2002.

12. Richards L. Handling qualitative data: A practical guide. 3rd ed. Los Angeles: Sage Publications; 2015.
