# Supplementary material for: Can you un-ring the bell? A qualitative study of how affect influences cancer screening decisions
Source: BMC Cancer. 2017 Sep 13;17:647. doi: 10.1186/s12885-017-3596-7 (PMC5598010; doi:10.1186/s12885-017-3596-7)
Supplement: Supplementary file 2 — Mammography Screening Focus Group Interview Guide. This document contains the semi-structured focus group instrument that guided discussions with women about mammography screening. The document also contains a graphic that was handed out to participants at the mid-way point of the focus groups that illustrates the number of women needed to be tested to prevent one death. Source of graphic was the Canadian Task Force on Preventive Health Care. “Recommendations on screening for breast cancer in average-risk women aged 40-74 years.” Canadian Medical Association Journal, November 22, 2011. (PDF 198 kb) [file 12885_2017_3596_MOESM2_ESM.pdf]

Mammography Screening Focus Group Interview Guide

General Opening Questions

Please state your first name and tell us how long you have lived here.

Off the top of your head, please write down what the first thing you think of when you think about breast cancer screening?

> Okay. Let's go around the table to hear people's responses, and don't be shy if your answer has already been read out.

Off the top of your head, please write down the first thing you think of when you think about population-based screening. Don't be afraid if you don't know what it is. If you are unsure, just write that down. (*Here we are just trying to see if people understand the difference between population-based screening from individual patients who might ask their doctors to have a mammogram if they are under the age of 50 – no jurisdiction has a policy to "ban" screening under age 50; these decisions are left to the discretion of the physician)*

> So far we've heard [list the cancer screening options already named]. Is there anything else women can do to catch it early if they do have breast cancer?

When you think about breast cancer, are there things that you're not sure about?

> *Probes:*
> What causes it, the risk of getting it, screening tests, treatments.

We hear lots of information about things that are good for us, other things that are bad for us. I always pay attention to the ones that say chocolate is good for you. What do you do when you hear those kinds of things?

> *Probes:*

Have you ever changed anything you do because of something you've heard in the news about health? Stopped doing something? Started something new? Doing something more or less often?

<u>Communication/Understanding about Cancer Control Case Scenarios</u>

We would like to ask you some questions about breast cancer screening, information you received or sought out, how good you thought that information was, and what you understood from the information you received.

What do you know about mammography?

A mammogram is an x-ray picture of the breast. Screening mammograms are used to check for breast cancer in women who have no signs or symptoms of the disease. Diagnostic mammograms are used to check for breast cancer after a lump or other sign or symptom of the disease has been found.

Have you or someone you know had a mammogram?

For participants who have had a mammogram:
Do you think you were told everything about the benefits and risks of having a mammogram?

- From your health care provider?
- Did you look for information on your own?

How much did you trust the information that you found? Why/why not?

Were you confused by any of the messages that you heard?

- Timing – when to start, how often to do it when you start, should the frequency of testing change as you get older
- Risks

- Benefits

What kind of information would you like to know about mammography?

How easy has it been to find information about mammography?

If you want to learn something about your health, or a member of your family's health, about something like breast cancer screening, where do you go for information?

- Media (newspapers, including online sources, radio, television, programs like Dr. Oz, The Doctors)
- Internet (like government websites, general health sites (official or unofficial)
- Social networking sites (Facebook, MySpace, Twitter, blogs)
- HealthLinks / Telehealth
- Your doctor or other health professionals
- Friends or family
- Others?

    - How much did you trust that source of information? Why/why not?

    - Were you confused by any of the messages that you heard?

        - Timing – when to start, how often to do it when you start, should the frequency of testing change as you get older
        - Risks
        - Benefits

Now that we have spoken a bit about some of these issues more generally, let's get into something that has been more recently discussed.

What have you heard in the news, if anything, about breast cancer screening in the last year?

NOTE: Distribute print copies of graphic tools illustrating current evidence about breast cancer screening.

Recently, the Canadian Task Force on Preventive Health Care has reaffirmed its consensus statement that mammography screening at a population level for otherwise healthy women begins at the age of 50 (i.e. women without a family history breast cancer or recent symptoms (e.g. lump) or other abnormalities in the breast). This has been a long standing policy position federally in Canada, even though some provinces like British Columbia do population based screening starting at age 40, but BC might be reconsidering their positions in light of the national task force recommendations. Similarly, in 2009 the US Preventive Services Task Force, raised its recommended age for population based screening using mammograms to the age of 50. The previous US position had been to begin screening at the age of 40. Despite this policy change, many States have not been able to implement this change due to public pressure against the move. This diagram shows some of these shifts (cool graphic will be shown that summarizes the risks/benefits of using age 40).

One of the big issues here is that some of the scientists and doctors who disagree about the best age to start having population based mammograms have different perspectives: Some doctors put more emphasis on the image and what they think will give them a clearer baseline for future testing, but other doctors aren't sure that this is true. On top of that, some doctors think that finding a cancer through screening *in this age group* – who are usually pre-menopausal, and knowing that hormones have a significant influence on the development and growth of breast cancers – won't change the available treatment options or the effectiveness of treatments, or necessarily improve a woman's quality of life, and it might expose women to unnecessary risk (including additional testing and anxiety).

For women in the 40-49 age group, finding a cancer through screening mammography and finding a cancer through symptoms or finding lumps – it isn't clear if it will lead to better outcomes.

- What do you think of the Canadian position?

- Do you have concerns over keeping the population-based age for routine mammography screening at age of 50? (why/why not)

- Do you think that Manitoba/Ontario should adopt a position closer to what is done in BC? (why/why not)

If you were to prioritize different ways of identifying breast cancer, which ones do you think are best at letting you and your doctor know that something is wrong?

Concluding questions

What would you hope policymakers would be thinking about in making decisions about things like breast cancer screening where there is still disagreement whether or not it works or is safe?

Are you worried that this takes away available resources from other viable health pursuits (if pop-based screening was lowered to age 40)?

How should society make decisions about balancing how health dollars are spent?

Any final comments that you would like to make?

Mammogram Screening in Women 40-49*

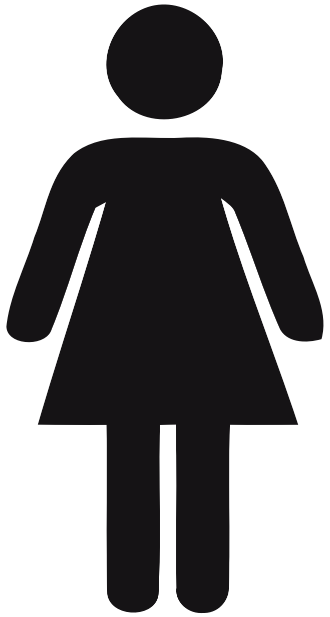

To prevent
1 Death

=

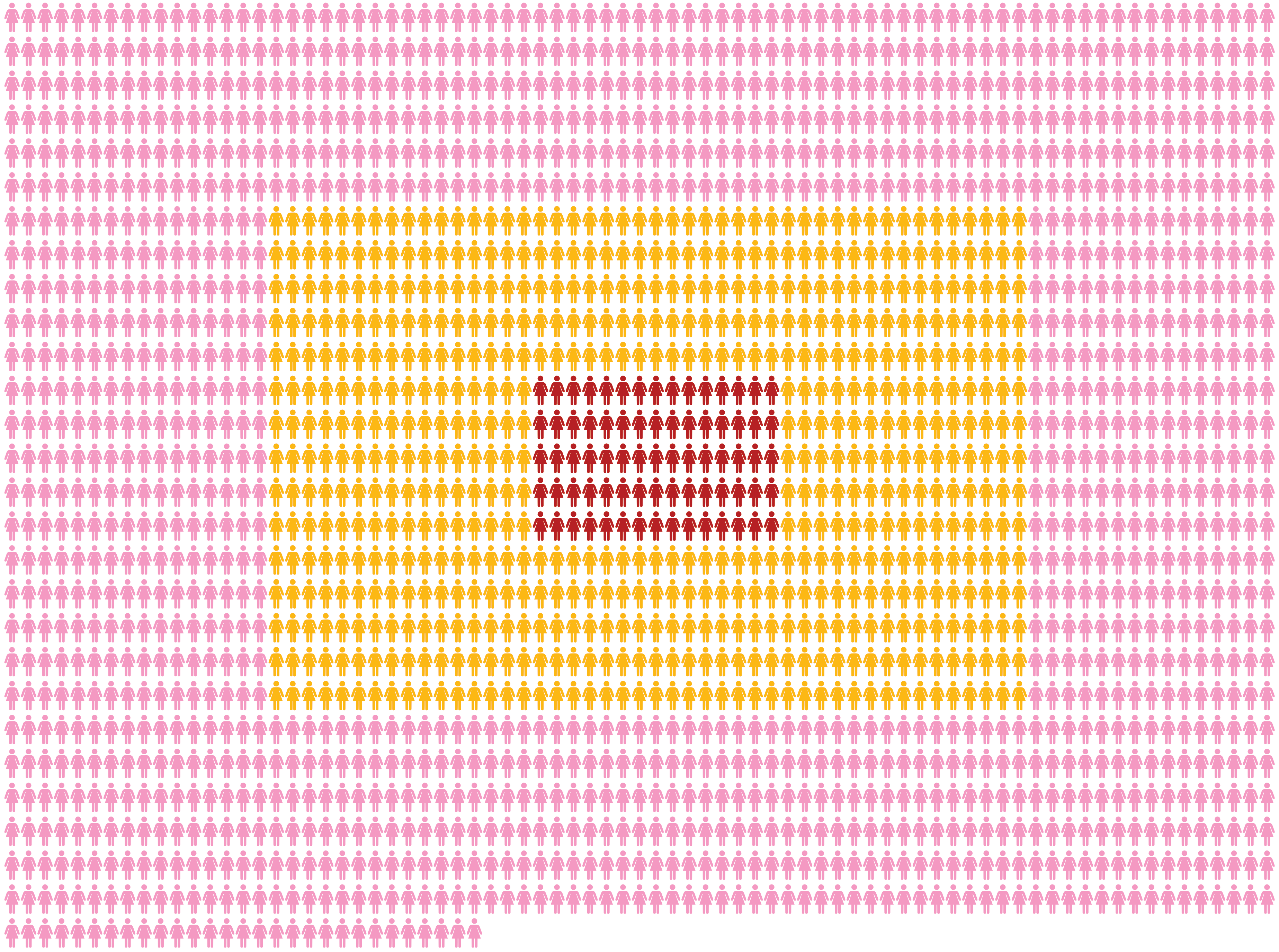

2108
women would have to be tested every two to three years for a total of 11 years

690
would have at least one false positive result

75
would have unnecessary biopsies

**Source: The Canadian Task Force on Preventive Health Care. "Recommendations on screening for breast cancer in average-risk women aged 40-74 years." Canadian Medical Association Journal, November 22, 2011.*

Mammogram Screening in Women 50-69*

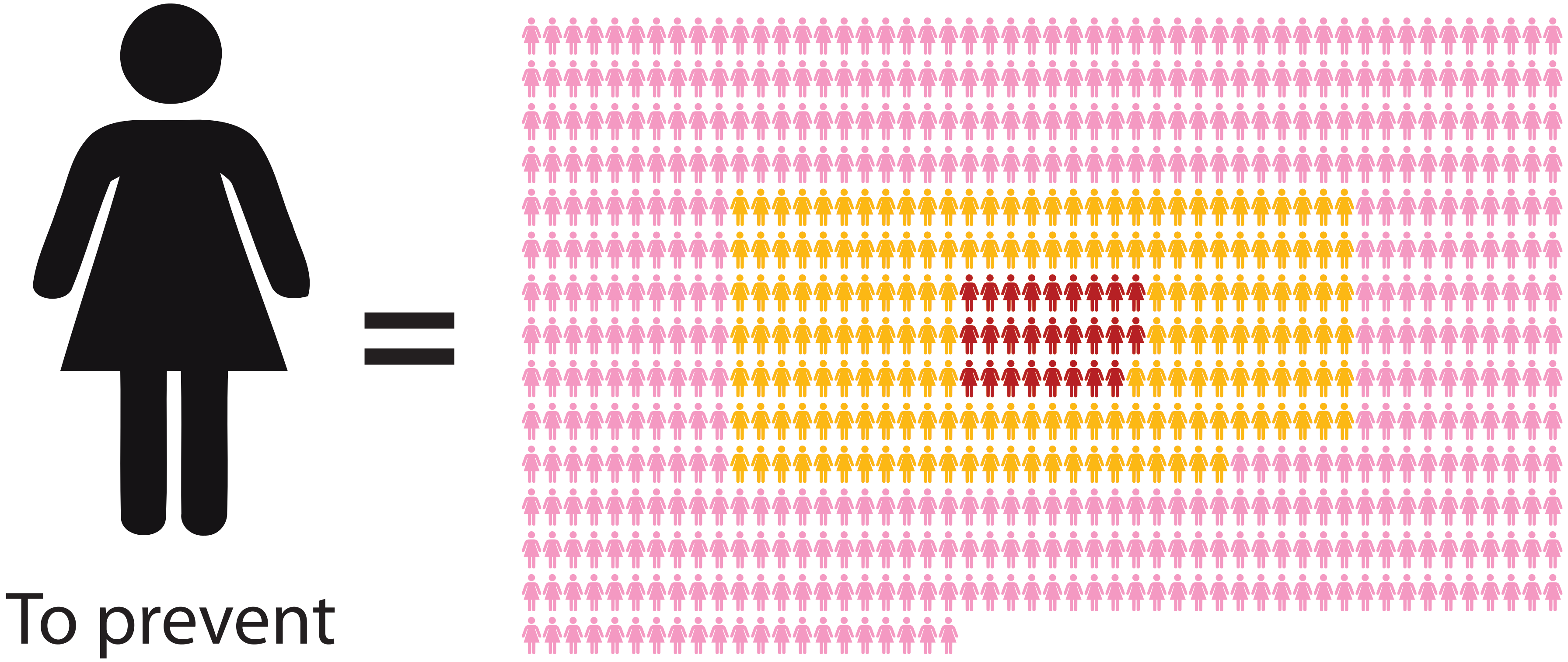

To prevent
1 Death

721
women would have
to be tested every
two to three years
for a total of 11 years

204
would have at
least one false
positive result

26
would have
unnecessary
biopsies

**Source: The Canadian Task Force on Preventive Health Care. "Recommendations on screening for breast cancer in average-risk women aged 40-74 years." Canadian Medical Association Journal, November 22, 2011.*
