# Supplementary material for: Can you un-ring the bell? A qualitative study of how affect influences cancer screening decisions
Source: BMC Cancer. 2017 Sep 13;17:647. doi: 10.1186/s12885-017-3596-7 (PMC5598010; doi:10.1186/s12885-017-3596-7)
Supplement: Supplementary file 3 — Focus Group Guide – Prostate Cancer Screening. This document contains the semi-structured focus group instrument that guided discussions with men about prostate cancer screening. The document also contains a handout that was distributed to participants at the mid-way point of the focus groups with summarizes the current research about prostate cancer screening. (PDF 149 kb) [file 12885_2017_3596_MOESM3_ESM.pdf]

Focus Group Interview Guide – Prostate Cancer Screening

<u>General Opening Questions</u>

Please state your first name and tell us how long you have lived here.

Off the top of your head, please write down what the first thing you think of when you think about testing for prostate cancer? (prompts if needed - PSA and DRE)

> Okay. Let's go around the table to hear people's responses, and don't be shy if your answer has already been read out.

Has anyone else heard about other testing options?

> *If they say yes (there are not).*

When you think about prostate cancer, are there things that you're not sure about?

> *Probes:*
> What causes it, the risk of getting it, screening tests, treatments.

We hear lots of information about things that are good for us, other things that are bad for us. I always pay attention to the ones that say chocolate or red wine is good for you. What do you do when you hear those kinds of things?

> *Probes:*
> Have you ever changed anything you do because of something you've heard in the news about health? Stopped doing something? Started something new? Doing something more or less often?

<u>Communication/Understanding about Cancer Control Case Scenarios</u>

We would like to ask you some questions about prostate cancer screening, information you received or sought out, how good you thought that information was, and what you understood from the information you received.

What do you know about the PSA test? (*Note: PSA = Prostate-Specific Antigen*)

A PSA serum level test measures the amount of prostate-specific antigen in the blood. This test is ordered to address a variety of problems of the prostate; in particular it may be used to look for cancer, but may also be used to assess when the prostate gland is inflamed (prostatitis), to monitor changes in prostate cancer, to evaluate the effectiveness of prostate cancer treatment, and check for recurring prostate cancer.

Have you or someone you know had a PSA test?

For participants who have had a PSA test:
What information did your doctor give you about the PSA test?

Do you think you were told everything about the benefits and risks of the PSA test?

- From your health care provider?
- Did you look for information on your own?

How much did you trust the information that you found? Why/why not?

Were you confused by any of the messages that you heard?

- Timing – when to start, how often to do it when you start, should the frequency of testing change as you get older
- Risks
- Benefits

What kind of information would you like to know about PSA testing?

How easy has it been to find information about PSA testing?

If you want to learn something about your health, or a member of your family's health, about something like prostate cancer screening, where do you go for information?

- Media (newspapers, including online sources, radio, television, programs like Dr. Oz, The Doctors)
- Internet (like government websites, general health sites (official or unofficial)
- Social networking sites (Facebook, MySpace, Twitter, blogs)
- HealthLinks / Telehealth
- Your doctor or other health professionals
- Friends or family
- Others?

    - How much did you trust that source of information? Why/why not?

    - Were you confused by any of the messages that you heard?

        - Timing – when to start, how often to do it when you start, should the frequency of testing change as you get older
        - Risks
        - Benefits

Now that we have spoken a bit about some of these issues more generally, let's get into something that has been more recently discussed.

Up to this point, we've talked about prostate cancer *testing*. Can anybody explain to us what prostate cancer *screening* is, or how it differs from testing?

> If you go to your doctor because you find a lump somewhere, or you see some blood in your toilet, or you have some other symptom you think might be a sign that you have a health problem, like cancer, and your doctor does tests to find out, that's *testing*. Cancer *screening* is different. Screening is when there is an organized program that sets out to systematically test all people who are at risk of getting particular kinds of cancer. To be screened is a 'choice' on the part of the individual, but a screening program is an organized system that is designed to bring people into the doctor's office, for example, for screening. It might mean reminders, etc.. Most provinces have breast cancer screening programs where women start getting called in for mammograms after they turn 50. In Manitoba/Ontario, there is a colo-rectal cancer screening program for people between 50 and 74, so a few months after you turn 50 most people get a kit in the mail to test for blood in their stool/feces.

What have you heard in the news, if anything, about prostate cancer *screening* in the last year?

This is still a very active area of research. There have been two really big studies, which have both been randomized trials, known as the “best” or “gold-standard” study design in medical research, but they have had conflicting conclusions. This is not unusual in science – any one study doesn’t “prove” a finding, and we have to do many studies in different populations to really understand what is going on. But it does lead to a perplexing situation when two studies don’t agree and the scientists argue passionately why their study has the better results. In this case, one study said that PSA screening would save men’s lives and one study found that it wouldn’t change the overall mortality outcome. Tens of thousands of healthy men without prostate cancer participated in these studies.

NOTE: Distribute print copies of graphic tools illustrating current evidence about prostate cancer screening.

Some of the other issues that come into play here are that some prostate cancers grow so slowly that they do not grow enough to affect a man’s quality of life before he dies from some other cause. But other prostate cancers are fast growing, and doctors can’t be sure which ones will grow slowly and could be safely left alone, and which ones will grow fast and need to be treated aggressively. There’s also issue that the PSA test is not the most reliable or precise cancer test there is. There is a high rate of false-positives, which are test results that incorrectly indicate a man has prostate cancer when he really doesn’t. A lot of those guys have to have unnecessary biopsies (and a lot of worry) to find out that they really don’t have prostate cancer. If that’s not enough, there’s also the issue of the side effects of treatments for prostate cancer, things like erectile dysfunction and incontinence.

You may be able to get a PSA test by asking your doctor for one, but no Canadian provinces have prostate cancer screening programs to test all men who are old enough to be at risk of getting prostate cancer.

The problem above is (a) there is not system and (b) the crux of the controversy – if it ought to be. These are precisely the arguments we want to hear. Do they understand the difference in the quality, precisions, “informativeness” of the test versus mammography.

- What do you think of the Canadian provinces’ position?

- Do you have concerns about not having routine prostate cancer screening for men old enough to get it (why/why not)

- Do you think that Manitoba/Ontario should provide prostate cancer screening? (why/why not)

If you were to prioritize different ways of identifying prostate cancer, which ones do you think are best at letting you and your doctor know that something is wrong?

<u>Concluding questions</u>

What would you hope policymakers would be thinking about in making decisions about things like prostate cancer screening where there is still disagreement whether or not it works or is safe?

> Are you worried that this takes away available resources from other viable health pursuits?

> How should society make decisions about balancing how health dollars are spent?

Any final comments that you would like to make?

Issues to Consider about Prostate Cancer Screening

Key: PSA=Prostate Specific Antigen
ED=Erectile Dysfunction

Research has shown that most men, if they live long enough, will develop prostate cancer.

… For many of these men, they won't even feel any symptoms (i.e. the cancer will not be a bother to them).

…These men will probably die of something else, as the prostate cancer is a very unaggressive type.

While PSA testing increases detection of prostate cancer, it also detects things in the prostate that are not cancer (i.e. the test can give a false-positive)

…more men with no prostate cancer (but who may have a higher PSA level as shown through the test) will have to undergo unnecessary biopsies to make sure they do not have cancer.

…puts men through a lot of potential unnecessary worry.

If a man tests positive for prostate cancer, deciding the next steps for treatment are not easy.

…some prostate cancers grow so slowly that they won't affect a man's quality of life, and can be left alone…

… BUT doctors can't be sure which cancers will grow slowly, and which will grow fast, needing aggressive treatment.

… PSA testing can sometimes help doctors and men with prostate cancer to watch for changes in PSA levels. Changes will tell whether or not the cancer is growing faster or staying the same.

Are the health risks worth possibly unnecessary treatment?

…major surgery, radiation treatments, impotence (erectile dysfunction), incontinence (urinary and fecal), and more.
